# The benefits of clustering in TNF receptor superfamily signaling

**DOI:** 10.3389/fimmu.2023.1225704

**Published:** 2023-08-17

**Authors:** Éva S. Vanamee, Denise L. Faustman

**Affiliations:** ^1^Immunobiology Department, Massachusetts General Hospital, Boston, MA, United States; ^2^Harvard Medical School, Boston, MA, United States

**Keywords:** TNF receptor superfamily (TNFRSF), TNF signaling, receptor clustering, TNFR agonism and antagonism, signal amplification

## Abstract

The tumor necrosis factor (TNF) receptor superfamily is a structurally and functionally related group of cell surface receptors that play crucial roles in various cellular processes, including apoptosis, cell survival, and immune regulation. This review paper synthesizes key findings from recent studies, highlighting the importance of clustering in TNF receptor superfamily signaling. We discuss the underlying molecular mechanisms of signaling, the functional consequences of receptor clustering, and potential therapeutic implications of targeting surface structures of receptor complexes.

## Introduction

The TNF receptor superfamily (TNFRSF) comprises a diverse group of cell surface receptors involved in regulating immune responses, inflammation, and cell survival (1). Dysregulation of TNF signaling is implicated in various pathological conditions, including cancer, autoimmune and allergic diseases. Recent studies have highlighted the importance of receptor clustering in the activation and modulation of TNF receptor signaling. In this review, we will summarize the key findings of TNFRSF signaling, the benefits of clustering in TNFRSF function and its implications for therapeutics development.

## Receptor classification and mechanism of action

**Figure 1:**
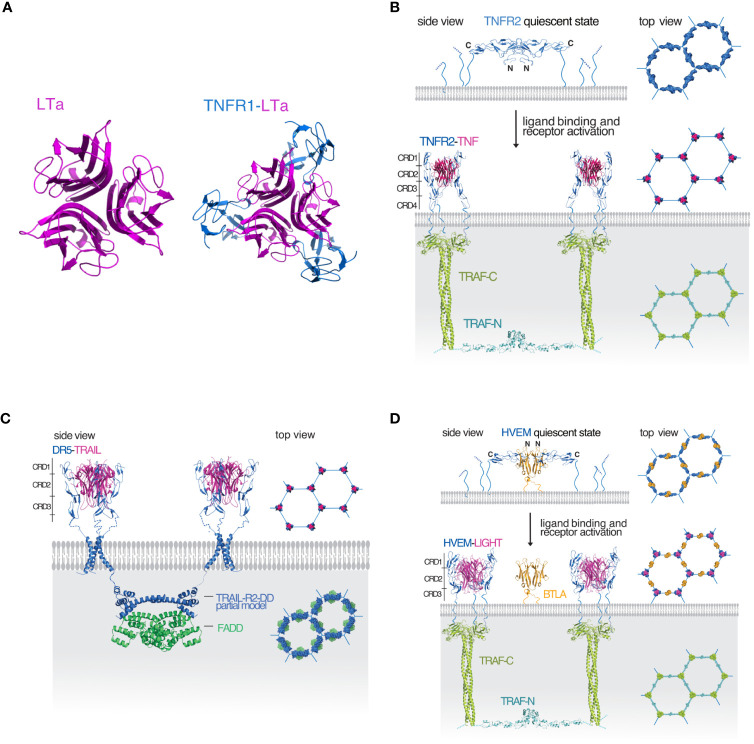
Illustration of the mechanism of signaling in the TNF receptor superfamily **(A)** Molecular representations of trimeric lymphotoxin (LTa) shown in magenta (top view) and the LTa (magenta)-TNF receptor 1 (TNFR1, blue) complex (top view). **(B)** A TRAF-interacting TNFRSF receptor represented by a model of the TNF/TNFR2/TRAF2 signaling complex. In the quiescent state (top panel), the receptor antiparallel dimers (blue) are arranged in a hexagonal lattice. TNF (magenta) binding breaks up the dimer interface and activated TNFR2 trimers recruit TRAF2 (green) resulting in the dimerization of the TRAF2 N-terminal RING domains (cyan) and activation of further downstream events. The hexagonal lattice of the downstream components mirrors the hexagonal lattice of the receptors. **(C)** Death receptor 5 (DR5, blue) in complex with its ligand TRAIL also forms a hexagonal cluster. After DR5 activation, TRAIL-R2-DD (blue) dimerizes and recruits a FADD dimer (green) also forming a hexagonal lattice. **(D)** For receptors like HVEM (blue) that are unable to dimerize on their own, hexagonal lattice formation is aided and controlled by the dimeric IgSF member, BTLA (orange). Upon binding of the ligand (magenta) the activated receptors recruit a TRAF homolog resulting in RING dimerization and activation of further downstream events. The program PyMOL was used for creating all molecular representations (3).

Members of the TNFRSF are type I, single pass membrane proteins with their C-terminal end anchored in the membrane. Their elongated ectodomains contain 1-6 cysteine rich domains (CRDs). TNF receptors can be grouped into three distinct groups (see **Table 1**): the first group contains receptors with a death domain (DD), essential for the initiation of apoptosis, though receptors in this group can also activate chronic inflammatory pathways. The second group of TNFRSF members interact with TNF receptor associated factors (TRAF) to initiate cell survival and proliferation via the canonical or non-canonical NFkB pathways. The third group contains decoy receptors that lack a functional cytoplasmic domain and instead act as decoys by binding to TNFSF ligands and prevent them from binding to other functional receptors. Ligands of the TNF superfamily (TNFSF) are type II membrane proteins with their N-terminal end anchored in the membrane. They share 20-30% sequence homology and a structurally conserved TNF homology domain (THD) (2). TNFSF ligands form non-covalent trimers and bind to three monomers of their corresponding receptors (**Figure 1A**). Efficient signaling in the TNFRSF requires that the receptors preassemble on the cell surface to form hexagonal honeycomb clusters (4–6). In return, the downstream signaling components assume the same hexagonal clustering geometry (7, 8). Free receptor monomers interact through the so-called pre-ligand assembly domain (PLAD) formed by the N-terminal and the CRD1 domains of the receptor (9–13). We have earlier proposed a uniform model that can apply to both the DD containing and TRAF-interacting receptors of the TNFRSF (4) (**Figures 1B, C**). To briefly summarize: the model assumes that receptors initially assume a quiescent state on the cell surface where trimers of antiparallel dimers form a hexagonal honeycomb cluster. The antiparallel dimer form partially buries the ligand binding surface and therefore unable to bind the ligand until activated. The activated receptors maintain the same clustering geometry and recruit the downstream signaling partners. The major benefit of this model is that the honeycomb lattice of the surface receptors is the same as the assembled honeycomb lattice of the downstream components so there is no need for major movement in the membrane upon activation. Since the downstream signaling partners form weak interactions, preassembly of the receptors on the cell surface enables the downstream partners to bind more efficiently once the receptors are activated by their ligands. This model accommodates both death receptors and TRAF-interacting receptors into one model.

**Table 1:** TNFRSF receptors, their ligands and intracellular binding partners.

TNFRSF Receptor (TNFRSF No.)	Number of CRD	Intracellular Binding Partner	TNFSF Ligand (TNFSF No.)	Receptor Stem Region (AA)
Death Receptors				
**TNFR1 (1a)**	4	TRADD, FADD, RIP	TNF (2), LTα (1), LTβ (3)	197-211
**Fas (6)**	3	FADD	FasL (6)	167-173
**TRAILR1 (10A)**	3^¶^	FADD, TRADD, RIP	TRAIL/Apo2L (10)	230-239
**TRAILR2 (10B)**	3^¶^	FADD, TRADD, RIP	TRAIL/Apo2L (10)	179-210
**NGFR (16)**	4	NADE	NGF (not a TNFSF member)	191-250
**DR3 (25 or 12)**	4^¶^	TRADD, FADD	TL1A (15), TWEAK (12)	193-199
**DR6 (21)**	4	TRADD, RIP	N-APP (not a TNFSF member)	212-349
**EDAR**	3^¶^	EDARADD	EDA-A1	149-187
Receptors with TRAF-interacting motif				
**TNFR2 (1b)**	4	TRAF1-3	TNF (2), LTα (1)	202-257
**LTβR (3)**	4	TRAF2-4, TRAF5	LTα (1), LTβ (3) as LTαβ_2,_, LTα_2_β	212-227
**OX40 (4)**	4^¶^	TRAF1-3, TRAF5, TRAF6	OX40L (4)	168-214
**CD40 (5)**	4	TRAF1-3, TRAF5, TRAF6	CD40L (5)	188-193
**CD27 (7)**	3	TRAF2, TRAF3, TRAF5	CD27L (7)	142-191
**CD30 (8)**	6	TRAF1-3, TRAF5	CD30L (8)	326-385
**4-1BB (9)**	4	TRAF1-3	4-1BBL (9)	160-186
**RANK (11A)**	4	TRAF1-3, TRAF5, TRAF6	RANKL (11)	195-212
**Fn14 (12A)**	1	TRAF2, TRAF6	TWEAK (12)	68-80
**TACI (13B)**	2	TRAF2-3, TRAF5, TRAF6	APRIL (13)	105-165
**BAFFR (13C)**	1	TRAF2, TRAF3, TRAF6	BAFF (13B/20)	36-78
**HVEM (14)**	3	TRAF1-3, TRAF5	LIGHT (14), LT-α (1)	163-202
**BCMA (17)**	1	TRAF1-3, TRAF5, TRAF6	APRIL (13), BAFF (13B/20)	42-54
**GITR (18)**	3	TRAF1-3	GITRL (18)	154-162
**TROY (19)**	3^¶^	TRAF1-3, TRAF5	LTα (1)	150-170
**RELT (19L)**	1	TRAF1	not known	110-162
**XEDAR (27)**	3^¶^	TRAF1, TRAF3, TRAF6	EDA-A2	119-138
Decoy receptors				
**TRAILR3 (10C)**	3^¶^	none	TRAIL/Apo2L (10)	
**TRAILR4 (10D)**	3^¶^	none	TRAIL/Apo2L (10)	
**OPG (11B)**	4	none	TRAIL/Apo2L (10), RANKL (11)	
**DcR3 (6B)**	4	none	FasL (6), TL1A (15), LIGHT (14)	

¶: Contains truncated CRD domains.

One potential issue with this model is that the number and size of the extracellular CRD domains of TNFRSFs vary and not all members have been shown to be able to form interactions via their PLAD. This raises the question whether the proposed model can hold for TNFRSF receptors that are unable to form stable dimers on their own. The answer has been provided by the structure of herpesvirus entry mediator (HVEM, TNFRSF14) in complex with the regulatory protein B- and T-lymphocyte attenuator (BTLA) (14).

## Immunoglobulin superfamily members aid TNFRSF clustering and regulate function

BTLA is the member of the Immunoglobulin (Ig) superfamily (IgSF) that comprises of proteins that play crucial roles in the immune system and other biological processes. These proteins are characterized by the presence of one or more Ig domains, which are structurally conserved regions that contain about 70-110 amino acids arranged in a sandwich-like structure (15). BTLA functions as an inhibitory receptor on T lymphocytes similar to well-known IgSF members such as cytotoxic T lymphocyte-associated antigen 4 (CTLA-4) and programmed death 1 (PD-1). BTLA interacts with the TNFRSF member HVEM that modulates B and T lymphocyte activation (16), dendritic cell proliferation (17) and protects mucosal epithelia from damage during inflammation (18). In the cytosol, HVEM interacts with several TRAF homologs including TRAF2 to induce NFkB activation. LIGHT and LT- α are the two canonical TNFSF ligands that activate HVEM. BTLA and CD160, another IgSF protein, regulate HVEM function.

The crystal structure of the HVEM-BTLA complex is a heterotetramer consisting of an antiparallel dimer of HVEM on the outside and a BTLA dimer on the inside (14) (**Figure 1D**). The complex closely resembles the antiparallel dimer structure of TNFR1 (19) and the modeled structure of TNFR2 shown in **Figure 1B**. Based on these data, we have earlier proposed that the HVEM antiparallel complex with BTLA may arrange in a hexagonal lattice on the cell surface representing the receptor quiescent state similar to TNFR1/2 (20). Since BTLA is a type I transmembrane protein similar to HVEM, both can be co-expressed and anchored to the cell by their C-terminal ends. In the *cis* configuration BTLA does not interfere with LIGHT or LT-α binding, instead it serves to facilitate HVEM oligomerization on the cell surface and to inhibit the ligand independent activation of HVEM. BTLA rather than being a true ligand of HVEM as previously proposed, serves as a regulatory protein modulating HVEM oligomerization and controls receptor activation. There are several TNFRSF receptors with three or fewer CRD domains that could potentially utilize a co-regulatory receptor to aid their oligomerization on the cell surface (see **Table 1**) and the HVEM/BTLA complex structure can serve as a template for such interactions.

We now have a unified model of TNFRSF that shows the receptors arranged in a honeycomb cluster both in their free and ligand bound states and this model can accommodate all members of the TNFRSF regardless of function or the size of their ectodomains. The size of the hexagonal lattice may vary from receptor to receptor but is expected to be the same for receptors that interact with the same downstream signaling partners. Next, we are going to discuss how the formation of the honeycomb cluster can improve signaling in the TNFRSF.

## Clustering enables cooperativity and leads to signal amplification

Cooperativity in the TNFRSF requires that at least two signaling trimers are placed close enough for an interaction to occur. Signal is transmitted first vertically after the binding of the ligand to the intracellular binding partners. For members of the TRAF- interacting receptors such as TNFR2 and HVEM, their cytoplasmic tails recruit TRAF homologs and it is TRAF dimerization via the N-terminal RING domains that enables cooperativity between signaling units (**Figures 1B, D**). TRAF binding proteins such as cIAP1/2 can also dimerize and facilitate cooperative signaling. In case of death receptors, such as CD95 (Fas) or death receptor 5 (DR5), their DDs recruit the Fas associated death domain (FADD) and it is FADD dimerization that enables cooperativity between two signaling units as seen in **Figure 1C**. In all instances, cooperativity requires that the two TNFRSF receptor trimers on the cell surface are activated by their ligands to create a logical AND gate.

We have shown earlier that cooperative signaling networks can be represented as planar graphs with nodes (***n***) and edges (***e***), where ***n*** represents the input signal and ***e*** the output signal (21). Cooperativity requires that at least two ***n*** input nodes are placed near each other at the right distance to create an ***e*** output. This generates one output signal from two input signals at a 50% loss of signaling efficiency. Even if thousands of TNFRSF signaling pairs are added onto the cell surface their efficiency remains at 0.5. However, if we order six input signaling units into a closed loop we end up with equal number of nodes and edges where ***e/n*** = 1. This closed signaling unit can be represented by a regular hexagon. Further clustering can then be illustrated by tiled regular hexagons. Tessellation or tiling refers to the process of covering a surface with one or more geometric shapes called tiles with no overlaps and no gaps. Mathematically, it means that the graph representing such system is a simple graph with no self-loops or multiple edges. A regular hexagon is one of only three regular polygons that can be tiled by themselves in two-dimension, the other two regular polygons are equilateral triangles and squares. As we have shown earlier, as the cluster size of tessellated polygons grow, the output/input signal ratio increases but can never exceed 3 (21). The maximum is also inversely proportional to the degree of the tiled polygon, therefore smallest in a hexagonal cluster and largest in a clustered system of tiled triangles where it can reach 300% of the original amplitude leading to the maximum value shown in Eq. 1, where **e** represents the sum of all edges and **n** represents the sum of all nodes in the cluster:


(1) $$\frac{output\;signal}{input\;signal}=\;\frac{e}{n}\leq \;3$$

The formula derived in Eq. 1 is the consequence of Euler’s polyhedron formula (22). It illustrates that the signal in a clustered network can be amplified. We have also shown that the amplification depends on cluster size and clustering geometry and it can broadly apply to all clustered cooperative signaling systems beyond the TNFRSF regardless of their molecular makeup (21).

**Figure 2:**
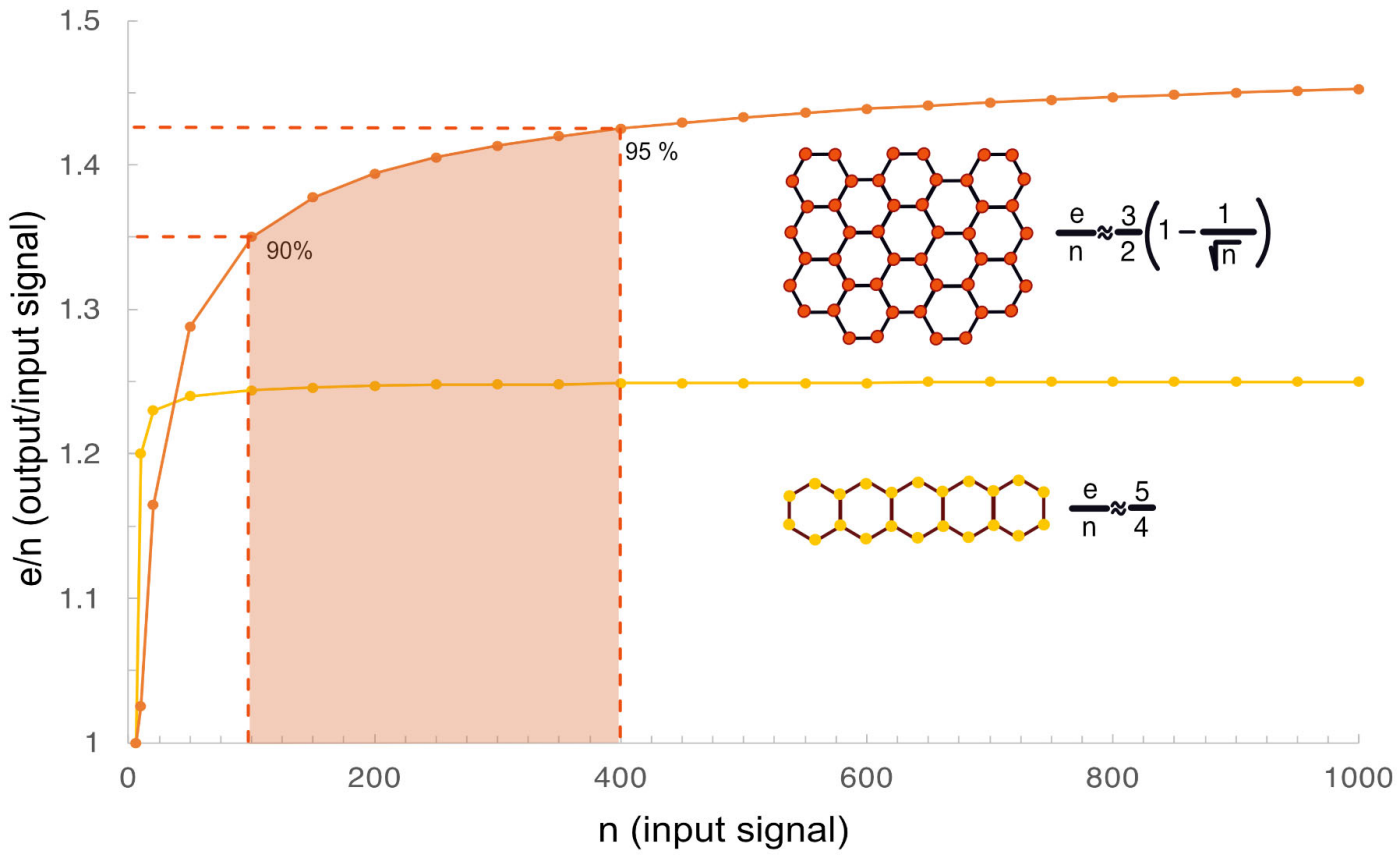
Illustration of signal amplification in hexagonal clusters. **(A)** Signal amplification represented by the **e/n** ratio is calculated for two examples of regular tiled hexagons with different geometries and plotted against **n**. It is higher in a hexagonal lattice that grows equally in both direction in the plane (shown in red) over a lattice tiled in only one direction (yellow). The red shaded area illustrates that 90-95% of the maximum signal amplification can be achieved with a cluster of 100-400 receptors in agreement with experimental data on the average size of receptor nano clusters in cells.

In **Figure 2** we provide examples of signal amplification in hexagonal clustering relevant to the TNFRSF. Ligand bound activated receptors represent the input signal that can be illustrated by the vertices or nodes. The dimerization of the TRAF RING domains or DD dimerization represent the output signal that is illustrated by the edges of the hexagon. The signal amplitude depends on the geometry of the honeycomb cluster. Tiling the hexagons in a more or less symmetrical fashion in each direction is the most efficient, leading to the highest **e/n** or input/output signal ratio compared to hexagons tiled in a linear fashion. This is because the **e/n** ratio is maximized when most hexagons are surrounded by other hexagons (21).

As the cluster size grows the **e/n** ratio increases and reaches a plateau. For hexagonal clusters 90% of the maximum signal amplification can be achieved in a cluster of 100 signaling units, and 400 units are required to reach 95% signal amplification. This is important because experimental data indicates that signaling receptors tend to form small clusters on the cell surface around 300-500 nm in diameter (23–25). Optimal cluster size may also dependent on the size of the cell-to-cell interface.

## Model of ligand activation of clustered receptors

**Figure 3:**
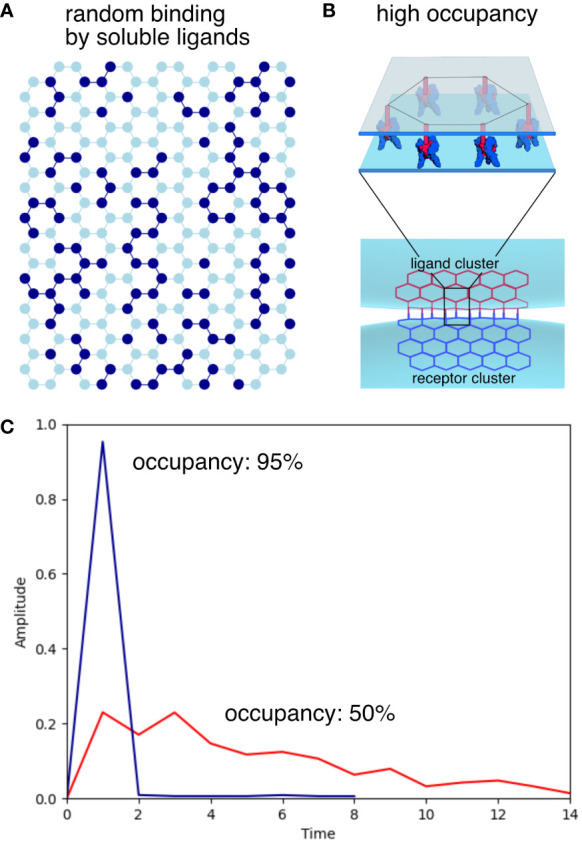
Illustration of simulated signal amplitudes with soluble or membrane-bound ligands. **(A)** Representation of a honeycomb receptor cluster with 50% occupancy. Activated receptors are represented as dark blue nodes, inactive receptors shown as light blue nodes. Edges between active nodes are shown in dark blue while all other edges are colored light blue. **(B)** In cell-to-cell interactions, clustered ligands attached to the membrane allow the simultaneous activation of clustered receptors with the same geometry, resulting in maximum signaling efficiency. **(C)** The amplitude is calculated as the ratio of active/inactive edges for 50% and 95% occupancies. High occupancy, illustrating membrane-bound ligand activation results in a sharp, high amplitude signal, while lower occupancy by soluble ligands results in a low amplitude signal spread out over time.

Now that we understand the optimal arrangement of receptor clusters on the cell surface, we can examine how ligand binding affects receptor activation and signaling. Ligands of the TNFSF are expressed as transmembrane proteins with their N terminal end anchored in the membrane. The ligands are cleaved to create a soluble form that is generally less effective than the membrane bound ligand across most of TNFSF. For instance, membrane bound TNF (memTNF) can activate TNFR2 very effectively but soluble TNF (sTNF) is a weak activator of TNFR2. We can illustrate how clustering can potentially explain these differences. **Figure 3A** illustrates a hexagonal lattice with receptor trimers represented as nodes in a hexagonal honeycomb grid. In this example, each activated (ligand bound) node is shown in dark blue, inactive nodes are in light blue, dark blue edges connect two active nodes, while all other edges are shown in light blue. In **Figure 3C** the amplitude (the ratio of the active edges over the total number of edges in the cluster) is calculated for 50% initial occupancy (red line) and 95% initial occupancy (blue line). The program then randomly activates a certain percentage of remaining inactive nodes until all are activated. When soluble ligands bind to their receptors, they bind more or less randomly with low overall amplitude. This is because even at 50% occupancy, not all activated receptor will be connected to other active trimers to create an output signal. When the ligands are bound to the membrane with the same geometry as the receptors (**Figure 3B**), the ligand trimers are going to line up with the receptor trimers and a much higher portion of receptors will be activated creating a strong signal similar to what is seen at high occupancy. The membrane bound ligands will generate a narrow and high amplitude, digital-like ON signal for activation. The soluble ligands on the other hand generate a low amplitude signal spread out over time as illustrated in **Figure 3C**. This is in remarkable agreement with experimental data using a DNA origami platform with immobilized FasL ligands arranged in different geometries to test the effect of ligand clustering on apoptosis efficiency in cells overexpressing the Fas receptor. Hexagonally arranged ligands generated a high amplitude signal in contrast to the low amplitude, broad signal generated by ligands with the wrong geometry (26). Super-resolution imaging has confirmed the importance of clustering *in vivo* in a Fas/FasL model but there is disagreement of the state of the ligand-free receptors (27). The Fas receptors appear largely monomeric and dimeric in the ligand-free state as observed by fluorescence energy transfer studies of C-terminal labeled Fas-fluorescence protein (Fas-FP) receptors. Fas-FP could appear monomeric even in the clustered state because the C-terminal ends of receptors in the ligand-free state maybe separated by a distance larger than the Förster distance of the FP pairs.

A low amplitude signal may not reach the threshold of activation and may result in not just quantitatively but qualitatively different signaling outcomes. This could explain how activation of the same receptor can result in different outcomes in the TNFRSF. Interestingly a recent paper on DR5 signaling provides an explanation of how the long isoform of the FLICE-like inhibitory protein (FLIP(L)) can act as both an inhibitor and promoter of caspase-8 at the death-inducing signaling complex (DISC) (28). The outcomes depend on the ratio of FLIP(L):caspase-8. When caspase-8 concentration is higher than FLIP(L) concentration apoptosis is accelerated. This can be explained by the proposed model of receptor activation of DR5 and the different outcomes generated by low vs high occupancy receptor clusters. Procaspase-8 binding to the DR5-DD-FADD complex activates caspase-8 and simultaneous activation of clustered DR5 with memTRAIL will lead to much higher concentrations of activated caspase-8 and a higher amplitude signal. Random activation of the receptor by sTRAIL could lead to much lower active concentrations of caspase-8 tilting the ratio in favor of FLIP(L) and result in apoptosis blockade.

FLIP has three functionally different isoforms. In addition to FLIP(L), two shorter isoforms FLIP(R) and FLIP(S) also exist and together they play a pivotal role in switching between cell survival, apoptosis and necroptosis. While FLIP(L) plays an important role in modulating apoptosis, FLIP(s) is important for assembling the necrosome to induce necroptosis. Necroptosis is the caspase independent regulated inflammatory form of cell death via cell lysis and necrosis. Receptor-interacting serine/threonine protein kinase-1 and 3 (RIPK1/3) and mixed lineage kinase domain–like (MLKL) are central mediators of TNF induced necrosis via TNF receptor 1 (TNFR1). Interestingly, TNFR1 internalization is an important first step in necroptosis (29). As we discussed earlier, receptor clustering and cluster stabilization can influence receptor internalization and therefore may also affect necroptosis. The ultimate outcome of cell fate is the result of a complex interplay of different cellular components and the activation or inhibition of several pathways. We believe that receptor clustering and the differential activation of clustered receptors play an important but not yet appreciated role in these processes.

**Figure 4:**
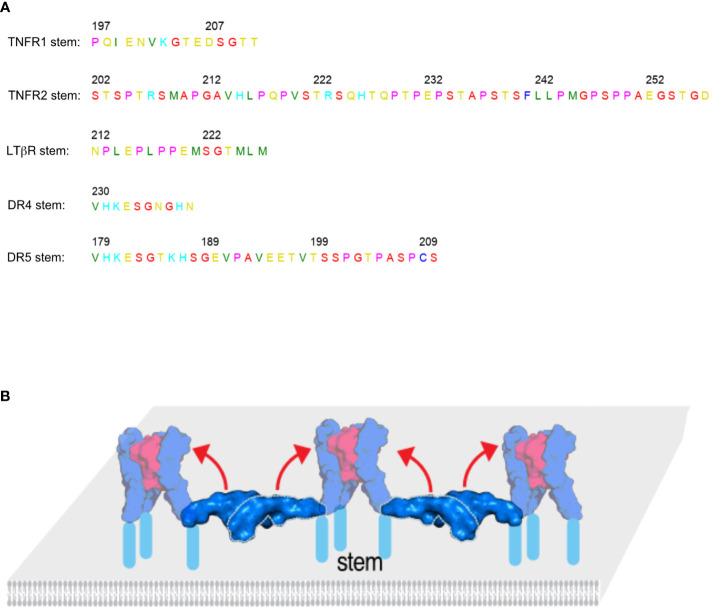
The size and rigidity of the stem region plays a role in receptor activation by the ligand. **(A)** The stem regions of several TNF receptors are listed. Soluble TNF can more readily activate TNFR1 and LTβR with short stem regions than TNFR2 that has a long stem sequence. Similarly, TRAIL can more readily activate DR4 with a short stem than DR5 that has a longer stem. **(B)** In the TNFR activation model the stem regions shown in light blue are exposed and may directly interact with the ligand or play an otherwise important role in receptor activation. The structures ligand bound complexes are shown in 50% transparency to indicate the final state after conformational change.

Clustering may not explain all the differences between membrane and soluble TNFSF ligands regarding their ability to activate the receptor. The stem (or stalk) region of the receptor plays an important role as well. In most cases, the stem region is defined by the sequence between the last CRD domain and the transmembrane domain of the receptor. When the stem regions of TNFR1 and TNFR2 are switched, sTNF can readily activate TNFR2 but not TNFR1 (30). TNFR1 has a short stem region (15 AA), while TNFR2 has a longer proline rich stem region (56 AA) (**Figure 4A****).** sTRAIL can also more easily activate DR4 that have a short stem region but not DR5 that has a longer stem (**Figure 4A**) (31). LTβR with a short stem region also belongs to receptors that are known to be readily activated by their respective soluble ligand. On the other hand, OX40, CD27, 4-1BB and TACI have longer proline rich stems and are less readily activated by their respective soluble ligands (30). **Table 1** lists the stem size for all TNFRSF members and there seems to be a clear correlation between stem size, rigidity and responsiveness to soluble TNFSF ligand. In the quiescent state model, trimers of antiparallel dimers setup the honeycomb cluster. The antiparallel dimer acts as a ruler to position receptor trimers at the right distance away from each other and to also sequester the ligand binding site. The dimer interactions are needed to create the right lattice of the honeycomb cluster. In the inactive, lateral state the stem region of each receptor is exposed and may directly interact with the TNFSF ligand (soluble of membrane bound) to initiate the conformational transition of the receptor from horizontal (inactive) to vertical (active) position (**Figure 4B**). This kind of transition is not unusual. In Munc13 that also forms hexagonal clusters, the Munc13 core (Munc13C) transitions between upright (open) conformation to lateral (closed) (32). We believe it warrants further research to address how the size and rigidity of the stem region may affect receptor activation by TNFSF ligands for other members of the TNFRSF.

We are now going to illustrate how taking into account the natural 3D structure of an antigen on the cell surface can guide the successful development of therapeutics with examples from the TNFRSF.

## Understanding the surface structures of the TNFRSF can aid the development of better therapeutics

It is important to highlight that early antibody development was done in the absence of high-resolution structures of the target antigen. Often, the exact mechanism of action of the therapeutic antibodies were unknown as well resulting in surprises decades later. Over the years research has shown that the target epitope can influence the function of antibodies and they can act both as agonists or antagonists. To untangle this relationship requires a more detailed understanding of the antigen structure and the relationship between the target epitope and antibody function. While several very successful anti-TNF therapeutics have been launched to treat rheumatoid arthritis, psoriatic arthritis and other autoimmune conditions (33), their mechanism of action still holds surprises even two decades after development. As an example, the anti-TNF antibody, adalimumab has only recently been shown to paradoxically function as a TNFR2 agonist (34). Adalimumab not only has been shown to bind to TNF but surprisingly to increase its expression on the surface of monocytes. As the authors wrote: “The mechanism that may underlie this surprising result is unclear, but one possibility is that adalimumab stabilizes membrane TNF at the cell surface and prevents recycling or cleavage to soluble TNF” (34). In the context of clustering, we propose that adalimumab may aid the formation and stabilization of TNF clusters on the cell surface that in turn may facilitate better signaling via the also clustered TNFR2 on the surface of cells. This is in agreement with experimental data showing the higher order complexes of anti-TNF antibodies in complex with TNF (35). Before this information became available, it was widely believed that TNF blockade and not TNFR2 activation was responsible for T_reg_ expansion. On the receptor side, it has been challenging to create therapeutic antibodies against the TNFRSF. Antibodies against the TNFRSF can either block signaling and function as antagonists or promote signaling to function as agonists. It is only during the last few years that we have begun to understand how antibodies binding to different epitopes and surface structures can achieve these opposing functions (4–6, 36, 37).

**Figure 5:**
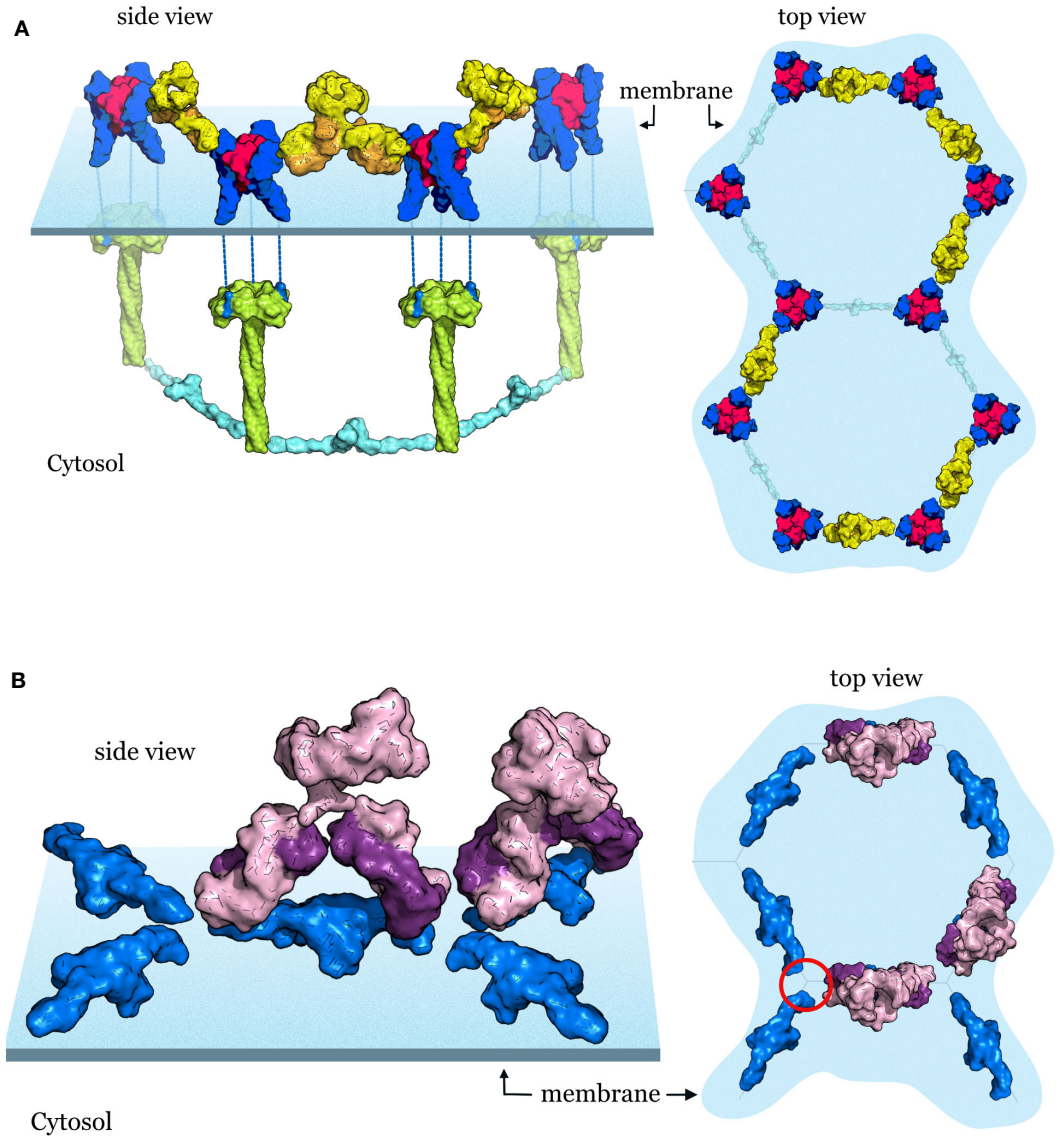
Antibody targeting strategies of TNF receptors **(A)** Agonist antibodies stabilize the hexagonal signaling complex TNF (magenta)-TNFR2 (blue) complexes are arranged on the cell surface in a hexagonal lattice. After receptor activation downstream TRAF signaling partners (shown in green and cyan) are recruited with matching hexagonal geometry. Agonist antibodies (shown in yellow and orange) stabilize the receptor clusters and improve signaling. **(B)** Antagonist antibodies stabilize the quiescent state and block the activation of receptors. Receptor dimers (blue) in the quiescent state are arranged in a hexagonal lattice on the cell surface. Antagonist antibodies shown in purple and violet lock in the ligand-free state and block ligand binding, receptor activation and the recruitment of downstream signaling partners.

For agonism, the stabilization of the hexagonal cluster of upright (free or ligand bound) receptors by antibodies that bind on the outside of the receptor, opposite the ligand binding site, may provide the best solution (6, 38) (**Figure 5A**). This strategy has been observed in an agonist antibody targeting DR5 (6) and also by an agonist targeting TNFR2 (38), highlighting that these strategies may be uniform regardless of the receptor type and their downstream partners. These antibodies link two receptor trimers together therefore both Fab arms are necessary. Stabilizing the receptor cluster may allow prolonged ligand binding and receptor activation or may directly activate the receptors in the absence of exogenous ligand. An additional potential benefit maybe the inhibition of receptor cleavage and/or receptor internalization. This seems to be the case in a recently developed artificial protein scaffold (39) that uses an inducible two-component system to produce hexagonal arrays to which receptors can be attached, thus allowing the study of geometry on signaling behavior. An important finding of the study is that the artificial protein scaffolds can modulate the internalization of the attached receptors with array size playing an important role in inhibiting endocytosis (39). A naturally occurring receptor also appears to employ this mechanism. In the epidermal growth factor receptor (EGFR) the transmembrane GxxxG motif plays an important role in oligomerization induced internalization and signal attenuation (40). The artificial 2D scaffold blocks receptor oligomerization and inhibits receptor internalization without inducing signaling (39), which could be important for therapeutic applications. Interestingly, several, but not all, TNFR members also contain the transmembrane GxxxG motif (41) that could play a similar role in modulating receptor internalization and signal attenuation in these members of the TNFRSF. Therefore, agonist antibodies against TNFRSF members that cross-link neighboring receptors greatly improve receptor stability and signaling (6, 38), and may inhibit receptor internalization by maintaining the separation of individual receptor trimers in the hexagonal lattice.

There are conflicting data in the literature on the need for Fcγ recruitment but it is not an absolute requirement for receptor agonism (4, 6, 38, 42–44). Neither the anti-DR5 nor the anti-TNFR2 agonist antibodies require Fcγ engagement for agonism (6, 38). Further examples include anti-CD40 and anti-Fn14 agonist antibodies that similarly do not require Fcγ engagement (45, 46). At times antibodies that function via Fcγ recruitment have been designated as agonists despite clearly blocking ligand binding. These antibodies should be more properly designated as therapeutics functioning via antibody dependent cell cytotoxicity (ADCC) and their function should be separated from true receptor agonism that does not depend on Fcγ involvement.

Historically, it has been difficult to create effective antagonist antibodies against the TNFRSF. For those working in the field, it is broadly appreciated by trial and error that the natural ligands to the TNFRSF not only have high affinity but also high avidity and can, in most cases, successfully compete against antagonist antibodies in challenge assays. For instance, antagonist antibodies raised against TNFR2 can block TNF binding with different effectiveness (36). Characterization of several antagonist antibodies to TNFR2 have shown that strong (or dominant) antagonists targeting the CRD3-CRD4 domain could effectively block TNFR2 signaling even in the presence of increasing concentrations of TNF, while weak (or recessive) antagonist antibodies target the CRD1-CRD2 domain and compete poorly with TNF (36). Further characterization revealed that, only the full antibody or the F(ab’)_2_ structure is able to successfully block TNF binding. The data supports a mechanism where the best antagonists bind to the antiparallel dimer form of the receptor locking in the non-signaling form of the receptor (4, 36) (**Figure 5B**). To highlight the case that structural homology can translate these findings to other members of the TNFRSF, an antagonist antibody to CD40 has also been shown to bind to the antiparallel dimer form of the receptor (47). In this case, the antibody binds as a single Fab domain making interactions to CRD1 of both CD40 monomers in the dimer. Interestingly, a mutation that abolishes binding to the antiparallel dimer form and results in the mutant antibody binding to a single CD40 monomer turns this antibody into a functional agonist proving that binding to the antiparallel dimer form is required for antagonistic activity (47).

Several groups have mapped the surface of TNF receptors to see how the epitopes influence function. As the above examples show, there is no clear connection between the epitopes position on the CRD and agonism or antagonism. However, the consensus that seems to be emerging is that the best agonists are bivalent or multivalent antibodies that cross-link and stabilize receptor complexes in the hexagonal cluster (6, 38) and the best antagonists are stabilizing the antiparallel form of the receptor (36, 47).

The antibody isotype can also have a huge influence on the function of antibodies both for agonism and antagonism. Several anti-CD40 agonist antibodies have been shown to benefit from isotype switching from the IgG1 to IgG2 isotype (48, 49). The IgG2 isotype has also improved the function of an anti-TNFR2 antagonist antibody (50). Structural and biophysical studies have shown that the IgG2 isotype is the most rigid of all the IgG isotypes with a narrower range in Fab movement and separation distance (51, 52). These studies suggest that both agonism and antagonism can benefit from the IgG2 isotype presumably by better stabilizing the hexagonal cluster with a less flexible antibody.

For ligand-based therapeutics targeting the TNFRSF, approaches that mimic the membrane-bound form result in much improved signaling. For a good review on different strategies see De Miguel et al. (53). The minimum requirement is the stabilization of the ligands by creating stable covalent trimers by various methods (54–56). This improves half-life and bioavailability. Generating a more stable and rigid ligand may also aid in the activation step of the receptors highlighted in **Figure 4B**. Linking two trimeric ligands can further improve signaling by activating neighboring receptors in the cluster (57–62). Several other ligand-fusion complexes have been created with improved signaling (53, 63–68). However, improvements in signaling by fusion constructs have to be carefully balanced against the risk of immunogenicity by unnatural looking complexes that the immune system may recognize as foreign. Indeed, many ligand constructs that have shown promise in the lab have never made it to the clinic for this reason.

## Conclusions

We have reviewed the current state of our understanding of TNFRSF signaling mechanism. We have shown that TNFRSF signaling can be described by a unified model that orders the receptors and ligands into a honeycomb cluster. The hexagonal lattice of TNF receptors is optimized for signal transduction as it provides the most economical way to build a stable scaffold. Clustering also results in signal amplification that depends on cluster size and geometry in agreement with experimental data showing TNF receptors are arranged in small nanoclusters on the cell surface. We have shown that high occupancy of a receptor cluster by ligands leads to a sharp, high amplitude signal, while random occupancy leads to broad low amplitude signal that is directly proportional to the concentration of RING dimers or caspase-8 generated and could explain differences in signaling outcomes between membrane and soluble TNFSF ligands. Building of more detailed signaling models in the future combined with experiments will further improve our understanding of the intricacies of TNFRSF signaling.

Beyond the TNFRSF, there are a growing number of hexagonal biological systems that indicate this may be a common arrangement of signaling networks in general. In addition to TNF receptors and their downstream signaling partners, chemo- or phototaxis receptors also cluster into hexagonal core complexes, consisting of trimers of dimers that further assemble to form large hexagonal arrays (69–71). Signal amplification has been observed in these systems and it has been proposed that the amplification is the result of cooperativity in the clustered arrays (71–73). Mimicking natural receptor clustering, artificial two-dimensional scaffolds have now been developed that utilize hexagonal lattices to modulate cell responses (39). The numerous available examples indicate the ordered clustering of surface proteins is more frequent in biological systems than previously appreciated, and most likely represents the rule and not the exception. It can also provide an optimal solution to the processing of biological information (21).

As the examples have shown a better understanding of receptor conformations on the cell surface can lead to the development of more effective therapeutics. Beyond antibody- and ligand-based therapeutics, the detailed knowledge of cell surface structures could also aid the development of small molecule drugs. Due to the high structural homology among members of the TNFRSF, strategies that work for the targeting of one receptor can be applied to others.

## Author contributions

EV and DF contributed to the design, writing and revision of the article. EV and DF approved the final content of the article.
